# Effects of COVID-19 measures on access to HIV/STI testing and condoms among adults in Sweden: a cross-sectional online survey

**DOI:** 10.1177/14034948231217020

**Published:** 2024-01-03

**Authors:** Maike Hentges, Anna E. Kågesten, Gunnar Brandén, Kyriaki Kosidou, Kristien Michielsen, Anna Mia Ekström, Elin C. Larsson

**Affiliations:** 1 Department of Global Public Health, Karolinska Institutet, Sweden; 2 Center for Epidemiology and Community Medicine, Region Stockholm, Sweden; 3 Institute for Family and Sexuality Studies, KU Leuven, Belgium; 4 Department of Infectious Diseases, South General Hospital, Sweden; 5 Department of Women’s and Children’s Health, Karolinska Institutet, Sweden; 6 Department of Women’s and Children’s Health, Uppsala University, Sweden

**Keywords:** COVID-19, sexual and reproductive health, HIV/STI testing, condoms, healthcare access, Sweden

## Abstract

**Aims:**

To investigate the self-reported impact of COVID-19 measures on access to testing for HIV and other sexually transmitted infections (STIs) and condoms and factors associated with reduced access among adults in Sweden.

**Methods:**

Cross-sectional data were collected in late 2020 through a web panel with adults (18-49 years) in Sweden as part of the International Sexual Health And REproductive health survey (I-SHARE) (*N*=1307). The primary outcome was self-reported access to HIV/STI testing and condoms during COVID-19 measures. Logistic regression was used to assess adjusted odds ratios of experiencing reduced access to HIV/STI testing and condoms in relation to sociodemographic characteristics, changes in sexual behaviours and COVID-19-related factors.

**Results:**

Of the 1138 sexually active respondents, 17% wanted an HIV/STI test, and of those over half (57%) reported reduced access during the COVID-19 measures in 2020. Compared with cis-women, transgender or non-binary respondents were more likely to experience lower access to testing. Among those who usually used condoms (*n*=568), 23% reported hampered condom access due to COVID-19 restrictions. Reduced condom access was associated with identifying as non-cis gender and a cis-man compared with cis-woman, non-heterosexual orientation, being foreign-born and financially worried.

**Conclusions:**

**Findings indicate that access to HIV/STI testing and condoms among sexually active adults of reproductive age in Sweden was disrupted during the COVID-19 pandemic in 2020 with varied impact depending on sexual orientation, gender identity or socioeconomic situation. This signals the importance of ensuring equitable access to sexual and reproductive health services and commodities in future crises response.**

## Background

In response to the spread of the severe acute respiratory syndrome coronavirus 2 (SARS-CoV-2) and associated disease (COVID-19), governments worldwide implemented population-level, non-pharmaceutical interventions, such as social distancing, school closures and travel restrictions – hereafter called COVID-19 measures – at different levels of stringency [1]. Sweden applied such measures, but without imposing a lockdown. Instead, the Swedish government, through the Swedish Public Health Agency, imposed strong recommendations for the population such as teleworking when possible, limiting social gatherings, avoiding unnecessary travel, public indoor environments and visits to elderly care, distancing enforcement in public transport and distancing learning for upper secondary and higher education [2]. The vast majority (90%) of the population followed these recommendations to a (very) high degree according to self-reported data in 2020 and 2021 [3]. However, growing evidence from different countries indicates that COVID-19 measures came with direct and indirect social, economic and health consequences, including for sexual and reproductive health (SRH) [4, 5]. Globally, emerging data across multiple income levels indicates that COVID-19 jeopardised equitable access to essential SRH services and commodities, including access to condoms and testing for HIV and other sexually transmitted infections (STIs) [6–8].

In 2020, HIV/STI testing providers across the globe reported their patients had disrupted access due to resource redistribution, clinic closures, transport limitations and reduced in-person services and laboratory capacities [6, 7]. Some groups were more affected than others, including young people, ethnic, sexual and gender minorities and those with lower socioeconomic status [7–11]. A global survey among men who have sex with men reported reduced testing access in parallel with stringent COVID-19 measures [12]. Although COVID-19 measures in Sweden were less restrictive compared with many other countries [2], the health system prioritised COVID-19, halting prevention programmes and shifting to more remote services [13].

Globally, there is a paucity of evidence on SRH service access including HIV/STI testing and condoms during the pandemic in the general population [8]. In response to this gap, the International Sexual Health And REproductive health (I-SHARE) project launched an online survey in 30 low-, middle-, and high-income countries to examine changes in SRH and sexual behaviours during the COVID-19 measures [14].

Using data from the Swedish I-SHARE survey implemented in late 2020, this study aims to investigate the self-reported impact of COVID-19 measures on access to HIV/STI testing and condoms among sexually active adults of reproductive age (18–49 years), and to assess factors associated with reduced access. We hypothesised that these measures impacted the accessibility of HIV/STI testing as well as condoms that normally is free of charge and is offered by various providers [15], thus potentially exacerbating previously identified barriers to HIV/STI testing and prevention [16].

## Methods

### Study design and population

I-SHARE Sweden collected cross-sectional data between 11 and 31 December 2020, targeting adults of reproductive age (18–49 years) across Sweden. A non-probability sample was recruited in Cint’s Swedish web panel aiming for 1000 responses based on available resources. The market research company Ipsos distributed survey invitations via email to eligible persons in the web panel. The selection aimed to obtain a representative sample of Sweden’s adult population concerning age (18–49 years), sex (men/women), and geographical location distributions (all regions), and to pay attention to representation of foreign-born adults. Ipsos sent about 10,000 opt-in survey links before meeting response quotas of the stratification variables (Supplemental Table I), retrieving 1307 respondents (i.e. 13% response rate). Respondents needed a digital device, provided online informed consent, and understood Swedish or English. Participants completed a self-administered, standardised online survey with single/multiple choice and free text questions about intimate relationships, sexual behaviours and access to SRH services and commodities 3 months before and after the initiation of COVID-19 measures, taking 15–20 min to complete [14]. The present analysis excluded 166 respondents without self-reported sexual experience alongside three respondents with missing values on sexual orientation, bringing the analytical sample size to *N*=1138.

### Measures

Primary outcomes included: (a) access to HIV/STI testing, assessed by ‘Have the COVID-19 measures stopped or hindered you from accessing a test for HIV or another STI?’ (yes/no); and (b) condom access, assessed by ‘Did the COVID-19 measures make it more difficult to access condoms?’ (no/yes/not applicable – I do not use condoms).

Secondary outcomes included access barriers to HIV/STI testing or condoms for those reporting difficulties (‘How did the COVID-19 measures stop or hinder you from accessing a test for HIV or another STI?’, ‘What made it difficult to access condoms?’). Respondents could choose multiple barriers to testing (e.g. no transport available/pharmacy or dispensary closed/health centre or clinic had long queues or is not accessible) and one main hindrance to condom access (e.g. no transport available/afraid I might acquire COVID/closed shops/condoms not in stock/not able or allowed to leave the house/pharmacy or dispensary closed/can no longer afford it). Those wanting to get tested were further asked which services they used or would use before and since COVID-19 measures (e.g. general practitioner/general hospital or clinic/HIV/STI clinic/online services/other).

Covariates included sociodemographic characteristics (age, country of birth, sexual orientation, area of residence, steady partner, gender identity), change in sexual behaviours (sexual activities and condom use with casual/steady partners since COVID-19 measures were introduced), and COVID-19-related factors (afraid of acquiring COVID-19, compliance with COVID-19 measures, financial worries during the pandemic). Gender identity was computed based on concordance between sex assigned at birth and gender the individual currently identifies with. Sexual behaviour questions were restricted to respondents with steady or casual partners in the three months before COVID-19 measures. Whether respondents had a steady partner at any point since the introduction of COVID-19 measures was identified based on multiple questions about relationship status before and since COVID-19 measures.

### Statistical analysis

Descriptive analysis was conducted for the distribution of variables. Pearson’s χ^2^ test compared proportions of primary outcomes per covariate to identify significant associations at α=0.05 (Supplemental Table II). χ^2^ or Fisher’s exact test examined differences between HIV/STI testing services used before versus after COVID-19 measures were introduced. Mann–Whitney tested for differences in median age between outcome groups. Multiple binary logistic regression analyses explored the adjusted odds ratios (aORs) of hindered testing and condom access, respectively, in relation to covariates. Models were stratified for sexual behaviour with steady versus casual partner to account for different sample sizes. Guided by the 10 events per variable (EPV ⩾10) criterion [17], we applied backward elimination with likelihood ratio tests to build models adjusted for age group, gender identity, sexual orientation, country of birth, financial worries and change in sexual activities and condom use with steady or casual partner. Covariates supported by previous research were kept irrespective of significance. The models’ fit was tested using the Hosmer–Lemeshow test. Multicollinearity was checked with a variance inflation factor (VIF) of less than 2. A two-tailed *P* value of less than 0.05 was considered statistically significant, and aORs are presented with 95% confidence intervals (CI). All analyses were conducted using Stata/SE version 17 (StataCorp LLC, College Station, TX, USA).

### Ethics

Ethical approval was obtained by the Swedish Ethical Review Authority (Dnr 2020-03969). Online informed consent was provided after information on the study’s purpose, respondents’ rights and data privacy was provided. Contact information for SRH services and women’s shelters in Sweden were listed. No identifying personal data were collected.

## Results

The sample consisted of 1138 sexually active eligible respondents, with a mean age of 35 years (standard deviation (SD) 8.8), and an equal gender identity division between cis-women (48%) and cis-men (46%), while 6% identified as transgender, non-binary or other (Table I). The majority (80%) identified as heterosexual, while one in five (20%) identified as homosexual/bisexual/other, a significantly higher percentage than in the general population (*c*. 10%) [15, 18]. Approximately one in six were foreign-born (15%). About half reported having financial worries during the pandemic (51%) and fear of acquiring COVID-19 (54%). The majority (83%) reported being highly compliant with COVID-19 measures. A few indicated decreased condom use with their steady (11%) or casual partners (17%; excluding missing) during the pandemic restrictions.

**Table I.** Characteristics of analytical sample (*N*=1138) and categorisation.

Characteristics	Value	*n* (%)	Categorisation	*n* (%)
**Sociodemographic factors**				
Age	No original categorisation		18–24	184 (16.2)
			25–35	429 (37.7)
			36–49	525 (46.1)
Sexual orientation	Heterosexual	906 (79.6)	Heterosexual	906 (79.6)
	Asexual	58 (5.1)	Homosexual/bisexual/other	232 (20.4)
	Bisexual	84 (7.4)		
	Gay	26 (2.3)		
	Lesbian	16 (1.4)		
	Pansexual	20 (1.8)		
	Other/unsure	28 (2.5)		
Sex assigned at birth	Woman	569 (50.0)	Cis-woman^a^	546 (48.0)
	Man	566 (49.7)	Cis-man^a^	523 (46.0)
	Other	3 (0.3)	Transgender/non-binary/other	69 (6.1)
Gender the individual currently identifies with	Man	538 (47.3)		
	Woman	566 (49.7)		
	Both	21 (1.9)		
	Neither	8 (0.7)		
	Other	5 (0.4)		
Country of birth	No original categorisation		Sweden	970 (85.2)
			Other	168 (14.8)
Area of residence	Capital city	221 (19.4)	Urban/suburban	954 (83.8)
	City	354 (30.2)	Rural/other	184 (16.2)
	Suburb of city	149 (13.1)		
	Town	239 (21.0)		
	Remote/rural area	180 (15.8)		
	Other	4 (0.4)	4 (0.4)	
Steady partner during COVID-19 measures	No original categorisation		No	315 (27.7)
			Yes	823 (72.3)
**COVID-19-related factors**				
Afraid of acquiring COVID-19	Totally agree	215 (18.9)	Agree	617 (54.2)
	Agree	402 (35.3)	Neutral	283 (24.9)
	Neither agree nor disagree	283 (24.9)	Disagree	238 (20.9)
	Disagree	151 (13.3)		
	Totally disagree	87 (7.6)		
Compliance with COVID-19 measures	Not at all	34 (3.0)	Low	189 (16.6)
	A little bit	155 (13.6)	High	949 (83.4)
	A lot	535 (47.0)		
	Very strictly	414 (36.4)		
Worried about financial situation	Totally agree	250 (22.0)	Agree	580 (51.0)
	Agree	330 (29.0)	Neutral	246 (21.6)
	Neither agree nor disagree	246 (21.6)	Disagree	312 (27.4)
	Disagree	158 (13.9)		
	Totally disagree	154 (13.5)		
**Change in sexual behaviours before versus since COVID-19 measures**				
Steady partner: sexual activities since COVID-19 measures^b^	Decreased a lot	76 (6.7)	Decreased	204 (17.9)
	Decreased a bit	128 (11.3)	Unchanged	463 (40.7)
	Stayed the same	463 (40.7)	Increased	137 (12.0)
	Increased a bit	103 (9.1)	Missing (skip logic)	334 (29.4)
	Increased a lot	34 (3.0)		
	Missing (skip logic)	334 (29.4)		
Steady partner: condom use with since COVID-19 measures^b^	Decreased a lot	31 (2.7)	Decreased	86 (7.6)
	Decreased a bit	55 (4.8)	Unchanged	635 (55.8)
	Stayed the same	635 (55.8)	Increased	83 (7.3)
	Increased a bit	58 (5.1)	Missing (skip logic)	334 (29.4)
	Increased a lot	25 (2.2)		
	Missing (skip logic)	334 (29.4)		
Causal partner: sexual activities since COVID-19 measures	Decreased a lot	74 (6.5)	Decreased	139 (12.2)
	Decreased a bit	65 (5.7)	Unchanged	883 (77.6)
	Stayed the same	883 (77.6)	Increased	116 (10.2)
	Increased a bit	74 (6.5)		
	Increased a lot	42 (3.7)		
Causal partner: condom use since COVID-19 measures^c^	Decreased a lot	22 (1.9)	Decreased	60 (5.3)
	Decreased a bit	38 (3.3)	Unchanged	203 (17.8)
	Stayed the same	203 (17.8)	Increased	94 (8.3)
	Increased a bit	63 (5.5)	Missing (skip logic)	781 (68.6)
	Increased a lot	31 (2.7)		
	Missing (skip logic)	781 (68.6)		

a Cis gender describes same sex assigned at birth and gender the individual currently identifies with.

b Change in sexual activities and condom use with steady partner was restricted to those with a steady partner in the 3 months before COVID-19 measures (*n*=804). Of those, 783 (97.4%) had a steady partner during COVID-19 measures.

c Change in condom use with casual partner was restricted to those having had sex with a casual partner in the 3 months before COVID-19 measures (*n*=357).

### Access to HIV/STI testing

**Figure 1.**
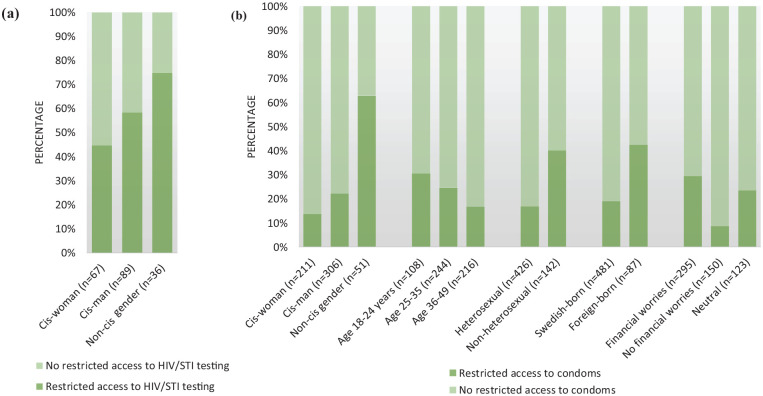
Percentage of adults with and without restricted access to (a) HIV/sexually transmitted infection (STI) testing and (b) condoms during COVID-19 measures by respondent groups.

One in six respondents (17%) reported wanting to take an HIV/STI test since the COVID-19 measures were introduced. Of those, more than half (57% CI 49.4–63.9) reported reduced access to testing. In bivariate analysis (Supplemental Table II), respondents identifying as transgender, non-binary or other were more likely than cis gender respondents to report reduced testing access (*P*=0.021) (Figure 1). Furthermore, respondents without disrupted testing access were more likely to report unchanged condom use with steady partners (56%) than changed use, while 36% versus 27% of those with lower access reported increased and decreased use, respectively (*P*=0.045). There were no significant differences in reporting restricted access to testing by age, sexual orientation, country of birth, area of residence, changed sexual activities and condom use with casual partner or COVID-19-related factors.

Table II depicts the aORs of hindered access to HIV/STI testing, stratified for changes in sexual behaviour with steady versus casual partners. Among respondents with steady partners (model 1), the only factor associated with HIV/STI testing access was gender identity; those identifying as transgender, non-binary or other were more likely to report hindered access compared with cis-women (aOR 3.30, CI 1.09–10.00). The same association was not significant for those with casual partners (Supplemental Table III).

**Table II.** Adjusted odds ratio of hindered access to HIV/STI testing and condoms due to COVID-19 measures in relation to covariates, by sexual behaviour with steady versus casual partners.

Covariate	Model 1: Hindered access to HIV/STI testing, steady partner (*n*=159)^a^			Model 2: Hindered access to condoms, steady partner (*n*=383)^b^			Model 3: Hindered access to condoms, non-steady partner (*n*=278)^c^		
	aOR	95% CI	*P* value	aOR	95% CI	*P* value	aOR	95% CI	*P* value
Age group (years)									
18–24	Ref			Ref			Ref		
25–35	1.81	0.77–4.29	0.175	0.94	0.49–1.80	0.855	0.96	0.53–1.73	0.890
36–49	1.59	0.64–3.93	0.319	0.69	0.34–1.40	0.301	0.80	0.42–1.51	0.485
Gender identity									
Cis-woman	Ref			Ref			Ref		
Cis-man	1.94	0.92–4.11	0.074	2.11	1.18–3.78	**0.012**	1.73	1.04–2.90	**0.036**
Transgender/non-binary/other	3.30	1.09–10.00	0.035	6.23	2.57–15.10	**<0.001**	4.82	2.15–10.81	**<0.001**
Sexual orientation									
Heterosexual	Ref			Ref			Ref		
Homosexual/bisexual/other	1.16	0.56–2.40	0.693	1.87	1.08–3.23	**0.025**	1.93	1.18–3.17	**0.009**
Country of birth									
Sweden	Ref			Ref			Ref		
Other	1.68	0.72–3.95	0.232	1.72	0.91–3.25	0.096	1.98	1.13–3.50	**0.018**
Worried about financial situation									
Disagree/neutral	Ref			Ref			Ref		
Agree	1.12	0.54–2.32	0.753	2.07	1.19–3.60	**0.011**	2.26	1.38–3.69	**<0.001**
Change in condom use with steady partner									
Decreased/unchanged	Ref			Ref			Ref		
Increased	1.95	0.80–4.77	0.143	0.70	0.41–1.20	0.192	0.67	0.41–1.09	0.104
Decreased/unchanged	.	.	.	*Ref*			*Ref*		
Increased	.	.	.	0.96	0.51–1.82	0.900	0.78	0.44–1.38	0.385

aOR: adjusted odds ratio; CI: confidence interval; Ref: reference category (aOR 1).

Bold *P* values denote significance at *P*<0.05.

a Model 1: Hosmer–Lemeshow test *P*=0.138; events per variable (EPV)≈11. aOR for change in sexual activities and condom use with steady partner, age group, gender identity, sexual orientation, country of birth, financial worries.

b Model 2: Hosmer–Lemeshow test *P*=0.095; EPV≈11. aOR for change in sexual activities and condom use with steady and casual partner, age group, gender identity, sexual orientation, country of birth, financial worries.

c Model 3: Hosmer–Lemeshow test *P*=0.174; EPV≈11. aOR for change in sexual activities and condom use with steady and casual partner, age group, gender identity, sexual orientation, country of birth, financial worries.

### Access barriers to HIV/STI testing

Participants who reported hindered access to testing (*n*=109) indicated multiple barriers. Postal services not functioning (41%, CI 31.9–51.1) was most common, interpreted as obstacles to mail HIV/STI self-tests, followed by closed pharmacies/dispensaries (28%, CI 19.4–36.9), inaccessible health centres/clinics (26%, CI 17.8–34.9) and unavailable transport (25%, CI 17.0–34.0). Fewer indicated HIV/STI testing services not being offered any more (7%, CI 3.2–14.0), not being able/allowed to leave the house (6%, CI 2.6–12.8), and other barriers (9%, CI 4.5–16.2).

### HIV/STI testing services

**Figure 2.**
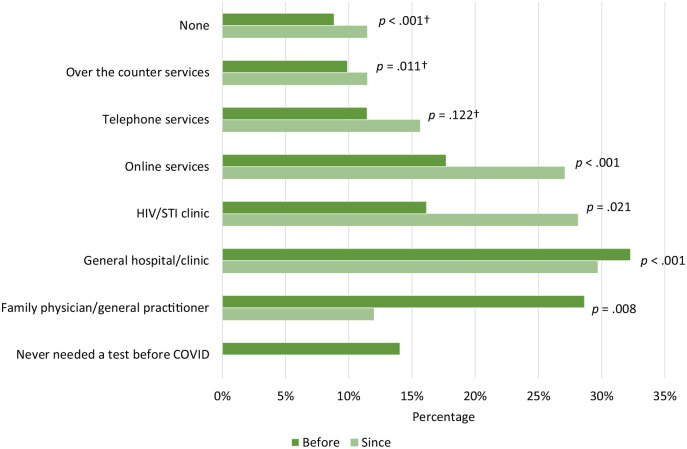
Percentage of HIV/sexually transmitted infection (STI) testing services used before versus since COVID-19 measures were introduced, *n*=192. Note. Multiple responses possible. P values derived from X² test. †Fisher’s exact test.

Respondents mostly reported using general hospitals/clinics to get tested for HIV/STI before COVID-19 measures (32%, CI 25.7–39.4), followed by general practitioners (29%, CI 22.4–35.6), online services (18%, CI 12.6–23.9) and HIV/STI clinics (16%, CI 11.2–22.1). Since COVID-19 measures, general hospitals/clinics were also most reported (30%, CI 23.3–36.7), followed by HIV/STI clinics (28%, CI 21.9–35.1), online services (27%, CI 20.9–34.0) and general practitioners (12%, CI 7.8–17.4). Respondents were less likely to use testing by general practitioners than before COVID-19 measures (*P*=0.008) and more likely to use online services (*P*<0.001) and HIV/STI clinics (*P*=0.021) (Figure 2).

### Access to condoms

Among participants who reported usually using condoms (*n*=568, 50%), approximately one in four (23%, CI 19.3–26.4) agreed that COVID-19 measures had hampered their access to condoms, and such agreement was independently associated with all covariates in bivariate models (Supplemental Table II). The Mann–Whitney U test showed a significant age difference (*P*<0.001) between those reporting more difficult condom access versus those who did not. On average, those reporting more difficult condom access were more likely than those who did not to be younger (18–24 years, *P*=0.012), foreign-born (29% vs. 11%, *P*<0.001), non-heterosexual (44% vs. 19%, *P*<0.001), and to identify as transgender, non-binary or other gender (25% vs. 4%, *P*<0.001) (Figure 1, Supplemental Table II). Reporting hindered condom access was less common among cis-women (23%) than cis-men (53%). There were also significant differences by area of residence (*P*=0.019), financial worries (*P*<0.001), having had a steady partner (*P*<0.001), changes in condom use and sexual activity with steady or casual partners (*P*<0.001), fear of acquiring COVID-19 (*P*=0.018) and compliance with COVID-19 measures (*P*<0.001).

Table II depicts the aORs of hindered access to condoms, stratified for changes in sexual activity with steady (model 2) versus casual partners (model 3). Among both groups, non-heterosexual adults were more likely to report reduced condom access compared with heterosexual adults (model 2: aOR 1.87, CI 1.08–3.23; model 3: aOR 1.93, CI 1.18–3.17). Financial worries during COVID-19 remained positively associated with hindered access (model 2: aOR 2.07, CI 1.19–3.60; model 3: aOR 2.26, CI 1.38–3.69). Followed by cis-men, non-cis gender individuals had the highest odds of hindered condom access compared with cis-women (model 2: aOR 6.23, CI 2.57–15.10; model 3: aOR 4.82, CI 2.15–10.81). Only among those with casual partners (model 3), foreign-born adults were more likely to report restricted access than Swedish-born (aOR 1.98, CI 1.13–3.50). Age, fear of acquiring COVID-19 and compliance with COVID-19 measures did not remain significantly associated with hindered condom access in the adjusted models.

### Access barriers to condoms

Respondents who reported hindered access to condoms (*n*=129) described closed shops (24%, CI 17.0–32.3), stock-outs of condoms (17%, CI 11.0–24.7), inability to leave the house (16%, CI 10.4–23.8), and fear of acquiring COVID-19 (16%, CI 10.4–23.8) as the main barriers. Less common barriers included closed pharmacies (9%, CI 4.3–14.8), unavailable transport (5%, CI 2.2–10.9), inaccessible health centres/clinics (5%, CI 1.7–9.9), unaffordability (5%, CI 1.7–9.9), restricted access to free condoms (1%, CI 0.02–4.2) and other barriers (2%, CI 0.5–6.7).

## Discussion

This study presents online survey results on HIV/STI testing and condom access among sexually active adults (18–49 years) in Sweden during the 2020 COVID-19 measures. Over half (57%) of respondents seeking testing during this time reported reduced access, and one in five who usually used condoms had difficulties accessing these (11% of total study sample). These estimates exceed the I-SHARE multi-country analysis, which found 32% reporting difficulties to access testing and 9% experiencing hindered condom access [19]. Our figures also exceed a cross-sectional survey in Britain, where 18% of men and 6% of women reported difficulties accessing condoms during lockdown [8]. While our results rely on self-reported data, they are substantiated by laboratory data from Sweden, indicating a decrease in STI tests in 2020 versus 2019 of up to 25% varying by region [20]. Similar reductions occurred in HIV/STI tests in Belgium [21], France [22] and the wider European region [6].

Our findings reveal intersectional inequities in HIV/STI testing and condom access. Sexual and gender minorities and cis-men were more likely to report restricted access than cis-women. This aligns with pre-pandemic evidence from Sweden showing that women were more likely than men to utilise STI testing, youth clinics and professional counselling [15, 23]. Possibly, cis-women in our study were also less affected if using alternative contraceptives in steady partnerships, reducing their need for condoms and opportunistic STI testing. French data also showed a greater reduction in STI testing for men than women during the pandemic [22]. Pre-pandemic evidence suggested that sexual and gender minorities faced barriers accessing HIV/STI testing and youth clinics in Sweden, indicating pre-existing inequities [24, 25]. Our research suggests that COVID-19 measures potentially intensified these inequalities, disproportionally affecting sexual and gender minorities and cis-men. In addition, foreign-born respondents and those reporting financial worries during the pandemic were particularly vulnerable to restricted condom access. While this intersectionality was less pronounced for testing access, this might be due to a smaller response group compared with condom access. Possibly, the shift towards digital SRH services [13], coupled with reduced drop-in visits and site/shop closures during peak COVID-19 measures may limit access for some groups (e.g. due to language barriers and lack of information about remote services). Reduced access to HIV/STI prevention and diagnosis can negatively impact transmission and treatment. Notably, respondents with restricted testing access were more likely to report increased condom use, indicating strong willingness to protect oneself against HIV/STI.

Our findings on intersecting disparities in accessing SRH services and commodities, based on gender identity, sexual orientation, socioeconomic and migration status, align with studies in high-income countries during the pandemic, such as the United States and Britain [9, 11, 12, 26]. Contrasting with prior research [8–10], young adults in our study were not more likely to report reduced testing and condom access after adjustment, possibly due to the increased use of remote services. Youth clinics may have also been less impacted than general clinics or practitioners that were likely to have shifted resources towards pandemic response. Unlike earlier literature [27], compliance with COVID-19 measures and fear of acquiring COVID-19 was not associated with hindered access in our study, highlighting how sociodemographic characteristics were likely to have mattered more than COVID-19-related factors.

Overall, our findings indicate that early COVID-19 measures in Sweden were likely to have exacerbated access difficulties. Inaccessible mail self-tests, pharmacies or health centres, and unavailable transport were key barriers to HIV/STI testing, while shop closures, stock-outs, inability to leave the house, and fear of acquiring COVID-19 restricted condom access. This reflects disruptions in service delivery, possibly indicating that health systems do not prioritise SRH services and commodities during health emergencies. Our study mirrors pre-pandemic issues with mail HIV/STI self-tests in Sweden [28], underlining the importance of maintaining access to in-person services when remote testing faces disruption. This is especially critical for those less likely to access such services.

### Strengths and limitations

To our knowledge, this study is among the first to investigate adults’ self-reported impact of COVID-19 measures on HIV/STI testing and condom access in Sweden. The web panel sample had representative age, sex and geographical distributions. The online survey was feasible for assessing self-reported pandemic effects on sensitive aspects of sexual health.

Nevertheless, self-reported data should be interpreted with caution due to potential bias (e.g. social desirability or recall bias). Our results are not generalisable to Sweden’s adult population. Non-response and selection bias in the web panel sample limit representativeness except in terms of age, sex and geographical location. The response rate was very low (13%), but the target sample size was reached by substituting persons who declined with ‘similar’ panelists on stratification variables. Regrettably, no information on non-responders was collected. The percentage of non-heterosexual respondents was higher than expected, while foreign-born adults were underrepresented (15% vs 20% in Sweden overall) [29], yet had higher representation than in most surveys. It was unknown whether respondents with steady partners also had casual partners, which could impact their SRH care needs. Therefore, we controlled for sexual behaviours instead of relationship status. Finally, the findings may reflect the pandemic’s general impact and service disruptions rather than the effect of response measures alone.

## Conclusions

Early COVID-19 measures in Sweden disrupted access to HIV/STI testing and condoms among sexually active adults of reproductive age – especially for sexual and gender minorities, cis-men, foreign-born adults, and those with worse economic situations during the pandemic. From an intersectional perspective, future crisis responses must ensure continuous and equitable access to SRH services and com-modities also in settings where less restrictive measures are implemented, as in Sweden. By maintaining access to in-person and remote services, pandemic responses may facilitate that everyone can enjoy their sexual experiences without risks to their health and wellbeing.

## Supplemental Material

sj-docx-1-sjp-10.1177_14034948231217020 – Supplemental material for Effects of COVID-19 measures on access to HIV/STI testing and condoms among adults in Sweden: a cross-sectional online survey Supplemental material, sj-docx-1-sjp-10.1177_14034948231217020 for Effects of COVID-19 measures on access to HIV/STI testing and condoms among adults in Sweden: a cross-sectional online survey by Maike Hentges, Anna E. Kågesten, Gunnar Brandén, Kyriaki Kosidou, Kristien Michielsen, Anna Mia Ekström and Elin C. Larsson in Scandinavian Journal of Public Health
