# Supplementary material for: Effects of COVID-19 measures on access to HIV/STI testing and condoms among adults in Sweden: a cross-sectional online survey
Source: Scand J Public Health. 2024 Jan 3;52(3):299–308. doi: 10.1177/14034948231217020 (PMC11067389; doi:10.1177/14034948231217020)
Supplement: sj-docx-1-sjp-10.1177_14034948231217020 – Supplemental material for Effects of COVID-19 measures on access to HIV/STI testing and condoms among adults in Sweden: a cross-sectional online survey [file sj-docx-1-sjp-10.1177_14034948231217020.docx]

# Supplemental material for “Effects of COVID-19 measures on access to HIV/STI testing and condoms among adults in Sweden: A cross-sectional online survey”

**Table I** Comparison of sex, age and geographical location division between the study sample, analytical sample, and target population.

|  | **Study sample**  **(N=1,307)** | **Analytical sample**  **(N=1,138)** | **Target population: Adults 18-49 years in Sweden, 2020** |
| --- | --- | --- | --- |
| **Covariate** | % | % | % |
| **Sex** |  |  |  |
| Man | 50.3 | 50.0 | 51.5 |
| Woman | 49.3 | 49.7 | 48.5 |
| Other | 0.5 | 0.3 | No data available |
| **Age** (years) |  |  |  |
| 18-24 | 18.4 | 16.2 | 19.1 |
| 25-35 | 37.0 | 37.7 | 37.8 |
| 36-49 | 44.6 | 46.1 | 43.2 |
| **Geographical area** |  |  |  |
| Blekinge län | 1.8 | 1.6 | 1.4 |
| Dalarnas län | 2.1 | 2.0 | 2.5 |
| Gotlands län | 1.0 | 1.0 | 0.5 |
| Gävleborgs län | 2.7 | 2.6 | 2.5 |
| Hallands län | 2.4 | 2.5 | 3.0 |
| Jämtlands län | 1.2 | 1.0 | 1.2 |
| Jönköpings län | 4.4 | 4.6 | 3.4 |
| Kalmar län | 1.8 | 1.9 | 2.1 |
| Kronobergs län | 2.1 | 2.3 | 1.9 |
| Norrbottens län | 2.2 | 2.5 | 2.2 |
| Skåne län | 12.5 | 12.8 | 13.5 |
| Stockholms län | 23.7 | 22.6 | 25.2 |
| Södermanslands län | 3.5 | 3.5 | 2.6 |
| Uppsala län | 3.0 | 2.9 | 4.0 |
| Värmlands län | 2.9 | 2.9 | 2.5 |
| Västerbottens län | 2.7 | 2.9 | 2.7 |
| Västernorrlands län | 2.5 | 2.6 | 2.1 |
| Västmanlands län | 3.0 | 2.6 | 2.5 |
| Västra Götalands län | 15.2 | 15.4 | 17.0 |
| Örebro län | 2.9 | 3.2 | 2.9 |
| Östergötlands län | 6.0 | 6.5 | 4.5 |
| Don’t know | 0.2 | 0.2 | No data available |

The target population data was retrieved in September 2023 from Statistics Sweden for the year 2020, available at: <https://www.statistikdatabasen.scb.se/>.

**Table II** Bivariate analysis of characteristics by hindered access to HIV/STI testing (n=192) and condoms (n=568) due to the COVID-19 pandemic.

| **Covariate** | **Hindered access to HIV/STI testing** | | | | **Hindered access to condoms** | | | |
| --- | --- | --- | --- | --- | --- | --- | --- | --- |
|  | **(n = 192)** | | | | **(n = 568)** | | | |
|  | **Yes**  *n* (col. %) | **No**  *n* (col. %) | **Total**  *n* (col. %) | ***P***  ***value*** | **Yes**  *n* (col. %) | **No**  *n* (col. %) | **Total**  *n* (col. %) | ***P***  ***value*** |
| **Sociodemographic factors** | | | | | | | | |
| Age group (years) |  |  |  | .954 |  |  |  | **.012** |
| 18-24 | 25 (22.9) | 20 (24.1) | 45 (23.4) |  | 33 (25.6) | 75 (17.1) | 108 (19.0) |  |
| 25-35 | 51 (46.8) | 37 (44.6) | 88 (45.8) |  | 60 (46.5) | 184 (41.9) | 244 (43.0) |  |
| 36-49 | 33 (30.3) | 26 (31.3) | 59 (30.7) |  | 36 (27.9) | 180 (41.0) | 216 (38.0) |  |
| Gender identity ^a^ |  |  |  | **.012** |  |  |  | **<.001** |
| Cis-woman | 30 (27.5) | 37 (44.6) | 67 (34.9) |  | 29 (22.5) | 182 (41.5) | 211 (37.1) |  |
| Cis-man | 52 (47.7) | 37 (44.6) | 89 (46.4) |  | 68 (52.7) | 238 (54.2) | 306 (53.9) |  |
| Transgender/Non-binary/  Other | 27 (24.8) | 9 (10.8) | 36 (18.8) |  | 32 (24.8) | 19 (4.3) | 51 (9.0) |  |
| Sexual orientation |  |  |  | .395 |  |  |  | **<.001** |
| Heterosexual | 63 (57.8) | 53 (63.9) | 116 (60.4) |  | 72 (55.8) | 354 (80.6) | 426 (75.0) |  |
| Homosexual/Bisexual/  Other | 46 (42.2) | 30 (36.1) | 76 (39.6) |  | 57 (44.2) | 85 (19.4) | 142 (25.0) |  |
| Country of birth |  |  |  | .207 |  |  |  | **<.001** |
| Sweden | 78 (71.6) | 66 (79.5) | 144 (75.0) |  | 92 (71.3) | 389 (88.6) | 481 (84.7) |  |
| Other | 31 (28.4) | 17 (20.5) | 48 (25.0) |  | 37 (28.7) | 50 (11.4) | 87 (15.3) |  |
| Area of residence |  |  |  | .397 |  |  |  | **.019** |
| Urban/Suburban | 101 (92.7) | 74 (89.2) | 175 (91.1) |  | 121 (93.8) | 378 (86.1) | 499 (87.9) |  |
| Rural and other | 8 (7.3) | 9 (10.8) | 17 (8.9) |  | 8 (6.2) | 61 (13.9) | 69 (12.1) |  |
| Steady partner during COVID-19 measures |  |  |  | **.016** |  |  |  | **<.001** |
| No | 11 (10.1) | 19 (22.9) | 30 (15.6) |  | 23 (17.8) | 155 (35.3) | 178 (31.3) |  |
| Yes | 98 (89.9) | 64 (77.1) | 162 (84.4) |  | 106 (82.2) | 284 (64.7) | 390 (68.7) |  |
| **COVID-19-related factors** | | | | | | | | |
| Afraid of acquiring COVID-19 |  |  |  | .472 |  |  |  | **.018** |
| Agree | 52 (47.7) | 45 (54.2) | 97 (50.5) |  | 57 (44.2) | 236 (53.8) | 293 (51.6) |  |
| Neutral | 35 (32.1) | 20 (24.1) | 55 (28.7) |  | 46 (35.7) | 102 (23.2) | 148 (26.1) |  |
| Disagree | 22 (20.2) | 18 (21.7) | 40 (20.8) |  | 26 (20.2) | 101 (23.0) | 127 (22.4) |  |
| Compliance with COVID-19 measures |  |  |  | .202 |  |  |  | **<.001** |
| Low | 34 (31.2) | 19 (22.9) | 53 (27.6) |  | 36 (27.9) | 67 (15.3) | 103 (18.1) |  |
| High | 75 (68.8) | 64 (77.1) | 139 (72.4) |  | 93 (72.1) | 372 (84.7) | 465 (81.9) |  |
| Worried about financial situation |  |  |  | .265 |  |  |  | **<.001** |
| Agree | 77 (70.6) | 52 (62.7) | 129 (67.2) |  | 87 (67.4) | 208 (47.4) | 295 (51.9) |  |
| Neutral | 20 (18.4) | 15 (18.1) | 35 (18.2) |  | 29 (22.5) | 94 (21.1) | 123 (21.7) |  |
| Disagree | 12 (11.0) | 16 (19.3) | 28 (14.6) |  | 13 (10.1) | 137 (31.2) | 150 (26.4) |  |
| **Change in sexual behaviours before versus since COVID-19 measures** | | | | | | | | |
| Steady partner: sexual activities since COVID-19 measures ^b^ |  |  |  | .653 |  |  |  | **<.001** |
| Decreased | 31 (31.0) | 21 (35.6) | 52 (32.7) |  | 31 (29.3) | 72 (26.0) | 103 (26.9) |  |
| Unchanged | 35 (35.0) | 22 (37.3) | 57 (35.9) |  | 32 (30.2) | 159 (57.4) | 191 (49.9) |  |
| Increased | 34 (34.0) | 16 (27.1) | 50 (31.5) |  | 43 (40.6) | 46 (16.6) | 89 (23.2) |  |
| *Missing (skip logic)* |  |  |  |  |  |  |  |  |
| Steady partner: condom use since COVID-19 measures ^b^ |  |  |  | **.045** |  |  |  | **<.001** |
| Decreased | 27 (27.0) | 14 (23.7) | 41 (25.8) |  | 29 (27.4) | 38 (13.7) | 67 (17.5) |  |
| Unchanged | 37 (37.0) | 33 (55.9) | 70 (44.0) |  | 36 (34.0) | 210 (75.8) | 246 (64.2) |  |
| Increased | 36 (36.0) | 12 (20.3) | 48 (30.2) |  | 41 (38.7) | 29 (10.5) | 70 (18.3) |  |
| *Missing (skip logic)* |  |  |  |  |  |  |  |  |
| Causal partner: sexual activities since COVID-19 measures |  |  |  | .119 |  |  |  | **<.001** |
| Decreased | 24 (22.0) | 16 (19.3) | 40 (20.8) |  | 32 (24.8) | 79 (18.0) | 111 (19.5) |  |
| Unchanged | 42 (38.5) | 44 (53.0) | 86 (44.8) |  | 48 (37.2) | 323 (73.6) | 371 (65.3) |  |
| Increased | 43 (39.5) | 23 (27.7) | 66 (34.4) |  | 49 (38.0) | 37 (8.4) | 86 (15.1) |  |
| Causal partner: condom use since COVID-19 measures *^c^* |  |  |  | .170 |  |  |  | **<.001** |
| Decreased | 23 (25.8) | 10 (24.4) | 33 (25.4) |  | 26 (23.9) | 28 (16.6) | 54 (19.4) |  |
| Unchanged | 25 (28.1) | 18 (43.9) | 43 (33.1) |  | 35 (32.1) | 115 (68.1) | 150 (54.0) |  |
| Increased | 41 (46.1) | 13 (31.7) | 54 (41.5) |  | 48 (44.0) | 26 (15.4) | 74 (26.6) |  |
| *Missing (skip logic)* |  | | | |  | | | |

*Note.* Bold *p*-values denote significance at p<.05.

^a^ Cisgender describes same sex assigned at birth and gender identity.

^b^ Change in sexual activities and condom use with steady partner was restricted to those with a steady partner in the three months before COVID-19 measures (*n*=804). Of those, 783 (97.4%) had a steady partner during COVID-19 measures. Denominators change to *n*=159 for access to HIV/STI testing and to *n*=383 for condom access due to the survey’s skip logic.

^c^ Change in condom use with casual partner was restricted to those having had sex with a casual partner in the three months before COVID-19 measures (*n*=357). Denominators change to *n*=130 for access to HIV/STI testing and to *n*=278 for condom access due to survey’s skip logic.

**Table III** Adjusted odds ratio of hindered access to HIV/STI testing due to COVID-19 measures in relation to covariates, by sexual behaviour with non-steady partner.

| **Covariate** | **Hindered access to HIV/STI testing, non-steady partner** | | |
| --- | --- | --- | --- |
|  | **(n=130) ^a^** | | |
|  | **aOR** | **95% CI** | ***p*** |
| Age group (years) |  |  |  |
| 18-24 | Ref |  |  |
| 25-35 | 1.78 | 0.67-4.76 | .248 |
| 36-49 | 2.41 | 0.78-7.51 | .128 |
| Gender identity |  |  |  |
| Cis-woman | Ref |  |  |
| Cis-man | 1.07 | 0.44-2.60 | .876 |
| Transgender/Non-binary/Other | 3.27 | 0.82-13.01 | .093 |
| Sexual orientation |  |  |  |
| Heterosexual | Ref |  |  |
| Homosexual/Bisexual/Other | 1.50 | 0.64-3.54 | .351 |
| Country of birth |  |  |  |
| Sweden | Ref |  |  |
| Other | 0.86 | 0.31-2.35 | .764 |
| Worried about financial situation |  |  |  |
| Disagree/Neutral | Ref |  |  |
| Agree | 1.10 | 0.48-2.54 | .816 |
| Change in condom use with steady partner |  |  |  |
| Decreased/Unchanged | . | . | . |
| Increased | . | . | . |
| Change in condom use with casual partner |  |  |  |
| Decreased/Unchanged | Ref |  |  |
| Increased | 1.66 | 0.67-4.11 | .277 |

*Note.* aOR=adjusted odds ratio. CI=confidence interval. *Ref*=reference category (aOR=1). Bold *p*-values denote significance at *p*<.05. ^a^ Hosmer-Lemeshow test *p*=.379; EPV$\approx$10. aOR for change in sexual activities and condom use with casual partner, age group, gender identity, sexual orientation, country of birth, financial worries.
